# A Review on Pulsed Laser Preparation of Quantum Dots in Colloids for the Optimization of Perovskite Solar Cells: Advantages, Challenges, and Prospects

**DOI:** 10.3390/nano14191550

**Published:** 2024-09-25

**Authors:** Liang Sun, Yang Li, Jiujiang Yan, Wei Xu, Liangfen Xiao, Zhong Zheng, Ke Liu, Zhijie Huang, Shuhan Li

**Affiliations:** 1 Department of Basic Courses, Naval University of Engineering, Wuhan 430033, China; 2 College of Electrical Engineering, Naval University of Engineering, Wuhan 430033, China; 3 Wuhan National Laboratory for Optoelectronics (WNLO), Huazhong University of Science and Technology (HUST), Wuhan 430074, China; 4 College of Electronic Engineering, Naval University of Engineering, Wuhan 430033, China; 5 Department of Information Countermeasures, Air Force Early Warning Academy, Wuhan 430019, China

**Keywords:** perovskite solar cells, quantum dots, pulsed laser irradiation in colloids, advantage, challenge and prospect

## Abstract

In recent years, academic research on perovskite solar cells (PSCs) has attracted remarkable attention, and one of the most crucial issues is promoting the power conversion efficiency (PCE) and operational stability of PSCs. Generally, modification of the electron or hole transport layers between the perovskite layers and electrodes via surface engineering is considered an effective strategy because the inherent structural defects between charge carrier transport layers and perovskite layers can be reshaped and modified by adopting the functional nanomaterials, and thus the charge recombination rate can be naturally decreased. At present, large amounts of available nanomaterials for surface modification of the perovskite films are extensively investigated, mainly including nanocrystals, nanorods, nanoarrays, and even colloidal quantum dots (QDs). In particular, as unique size-dependent nanomaterials, the diverse quantum properties of colloidal QDs are different from other nanomaterials, such as their quantum confinement effects, quantum-tunable effects, and quantum surface effects, which display great potential in promoting the PCE and operational stability of PSCs as the charge carriers in perovskite layers can be effectively tuned by these quantum effects. However, preparing QDs with a neat and desirable size remains a technical difficulty, even though the present chemical engineering is highly advanced. Fortunately, the rapid advances in laser technology have provided new insight into the precise preparation of QDs. In this review, we introduce a new approach for preparing the QDs, namely pulsed laser irradiation in colloids (PLIC), and briefly highlight the innovative works on PLIC-prepared QDs for the optimization of PSCs. This review not only highlights the advantages of PLIC for QD preparation but also critically points out the challenges and prospects of QD-based PSCs.

## 1. Introduction

As a natural energy, solar energy is clean and infinite. Making full use of solar energy to serve people’s daily lives is a desirable purpose of academic research and technical innovation. Over the past ten years, with the explosive development of the photovoltaic field, perovskite solar cells (PSCs) have attracted remarkable attention and been extensively investigated because of their superiorities in optical absorption efficiency, tunable bandgap, and outstanding carrier mobility, as well as their long charge diffusion length [1,2]. In the latest report, the power conversion efficiency (PCE) of PSCs has achieved a certified 26.54% (by NPVM), which exhibits the growing prospects of PSCs [3]. However, to accelerate the advance of practical applications, further promoting the PCE and operational stability of PSCs has evolved to be a fatal issue. In general, for PSCs with multi-layer structures, the weak light absorber and the fast recombination rate of charge carriers between the perovskite layer and electrodes have blocked the ultimate optimization of the PCE. Hence, in recent years, preventing and reshaping the inherent structural defects of the perovskite films by adopting nanomaterials or adding additive agents is considered an effective strategy because the charge carriers transported on the perovskite films can be confined by the defect states, and the defects may be passivated and even eliminated by the introduction of the various nanomaterials [4,5,6].

In previous reports, nanocrystals, nanorods, nanoarrays, and even quantum dots (QDs) are utilized to modify the structural defects of perovskite layers [7,8,9,10]. For instance, H. Wang et al. proposed a La-doped BaSnO_3_ (marked as “LBSO”) nanocrystal with bare surface states for embedding the grain boundaries of hybrid perovskite films. The defect states in grain boundaries that originated from the low-temperature film processing can be tailored via the laser-generated LBSO nanocrystals to improve the carriers’ dynamics and the environmental stability of PSCs. The LBSO nanocrystal provided an additional channel to facilitate effective carrier extraction and reduce carrier recombination, leading to a maximum PCE of 21.11% with negligible hysteresis for the mixed-cation PSCs [7]. C. Pelicano et al. prepared a highly crystalline ZnO nanorod by electrochemical deposition in a chloride medium and then constructed ZnO nanorods with charge-selective layers and a rubrene poly(3-hexylthiophene) (P3HT) bilayer (defined as “ZnO/P3HT”) for PSCs [8]. Under the synergistic effect and infiltration of ZnO nanorods, a shorter charge carrier path length can be obtained in perovskite layers, and a better photovoltaic performance of 4.9% is illustrated compared to the device with P3HT as the only hole-transporting layer. M. Chen et al. theoretically investigated the light absorption behavior of textured PSCs with two-dimensional TiO_2_ nanoarrays as an electron extraction layer by using the finite element method (FEM) [9]. The simulation results reveal that a higher light absorption efficiency of 64.8% for a 100 nm thick textured perovskite layer is achieved under air mass 1.5 global (AM 1.5 G) solar irradiation and an increase of 16.8% in comparison to the control planar cell. Furthermore, W. Chi et al. also discussed a series of issues in perovskite QDs for PSCs, including device fabrication with perovskite QDs, light absorption, charge transport, and their stabilities [10]. Compared with other bulk materials in the nanoscale, the quantum confinement effect, quantum-tunable effects, and quantum surface effects of QDs promote their suitability as an absorber in PSCs because the charge carriers in perovskite layers can be effectively tuned by these quantum effects, and the defects of perovskite layers can be properly modified. Thus, the fast recombination rate of charge carriers and the energy loss that originated from the structural defects in the interfaces between perovskite layers and electrodes can be effectively decreased by the synergistic effect of QDs. Meanwhile, towards the challenges of defect states from the processing procedure of hybrid perovskite films (which caused the interval destruction of each perovskite lattice unit), the morphology and surface states can be passivated by the small-sized QDs (<10 nm), and thus the PCE and operational stability of PSCs can be promoted.

Herein, as size-dependent low-dimensional nanomaterials (average size: 1~10 nm), QDs exhibit various properties for optoelectrical and biomedical applications [11]. Apart from the quantum effects introduced above, QDs for light-emitting diodes (LEDs), laser devices, PSCs, sensors, and quantum star-light sources at present have also attracted remarkable attention [12,13,14]. In particular, in 2023, innovative works in the QDs field were awarded the Nobel Prize in chemistry, which inspired more and more researchers to devote attention to the research and development of QDs [15]. In general, according to the different preparation methods, QDs can be classified as colloidal and epitaxial types [16,17,18]. Colloidal QDs refer to the tiny semiconductor particles dispersed in a stabilizing solvent. Compared with epitaxial QDs (nanoscale heterostructures embedded in a solid-state III–V semiconductor), colloidal QDs can be prepared by chemical and physical approaches at room temperature such as the hot injection method and pulsed laser fabrication in colloids, while epitaxial QDs are usually prepared by molecular beam epitaxy or metal-organic chemical vapor deposition on a semiconductor substrate under strict vacuum conditions [17,18]. Hence, colloidal QDs are much easier to prepare than epitaxial QDs and are widely used in LEDs, laser technology, cadmium-free emitters, PSCs, and lighting [19,20]. 

**Figure 1:**
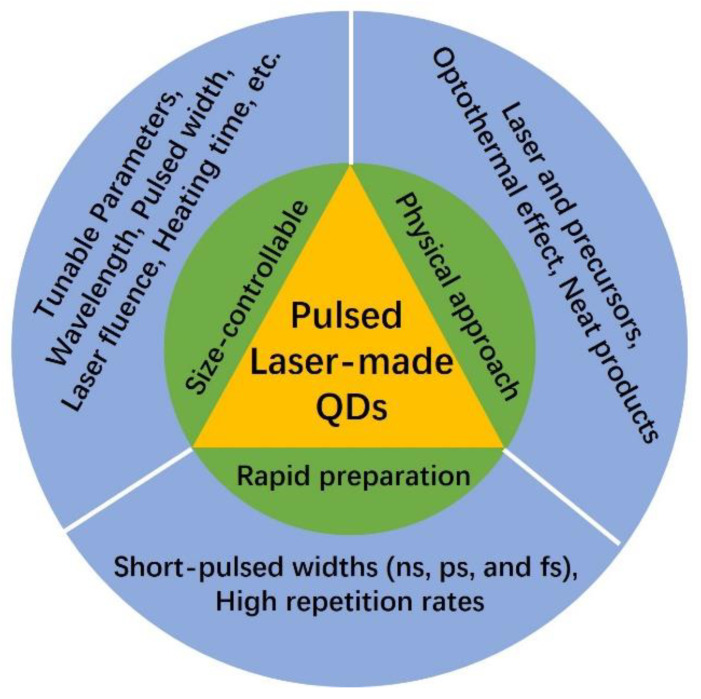
The advantages of pulsed laser-made QDs for PSCs.

To date, to modify the perovskite layers in PSCs, colloidal QDs, polymer nanomaterials, and organic small molecules are usually utilized [21,22]. Herein, as for the colloidal QDs, considering the nanoscale of perovskite structures, obtaining colloidal QDs with the desirable average size and neat purity is crucial for photovoltaic performance [21]. However, the conventional methods for colloidal QD preparation are generally wet chemical approaches, such as the typical hot injection method, and the QDs can be prepared by fabricating the precursors under suitable conditions [22]. During the hot injection of precursors, a series of complex chemical reactions assisted by different temperatures are required (including heating, cooling, and precipitation). Even though the QDs can be well-prepared, neat and size-controllable QDs are hard to realize because the QD products cannot be uniformly treated under the current conditions. Fortunately, with the rapid development of laser technology, pulsed laser irradiation in colloids (PLIC) has been utilized to fabricate the QDs. The PLIC technology was initially proposed and developed by the groups of P. Patil, P. Kamat, and N. Koshizaki, and lots of nanoscale or micrometer spherical particles were successfully prepared by PLIC [23,24]. Inspired by PLIC for nanoparticle preparation, using this method, X. Li et al. successfully prepared carbon QDs with visible and tunable photoluminescence (PL) in ordinary organic solvents [25]. The QDs were generated from the carbon nanomaterial-containing colloidal precursors, and the tunable PL property originated from the size-controlled QDs products fabricated under different laser fluences (300, 360, 420, and 480 mJ/pulse·cm^2^), which revealed that the QDs can be rapidly prepared using the PLIC method. In addition, H. Yu et al. also successfully fabricated carbon QDs with tunable PL by using pulsed laser non-focusing irradiation directly in a toluene solvent [26]. Different from the previous report, during laser irradiation, the organic toluene solvent not only served as the carbon source but was also utilized as the colloidal suspension for the formation of QDs. As shown in Figure 1, the advantages of PLIC for QDs used in PSCs mainly include rapid preparation, a physical approach, and size-controllable characteristics [25,26]. A pulsed laser with short-pulsed widths (ns, ps, and fs magnitudes) and high repetition rates can be rapidly heated in colloids so that the colloidal QDs can be rapidly prepared. As a laser heating approach, the colloidal QDs are generated from the optothermal effect between the laser beam and precursors (without using chemical agents), so the purity of laser-made QDs is very neat, which is completely different from the conventional chemical approach (usually assisted by chemical groups). In addition, because the temperature between the laser beam and heated precursors can be well-controlled by the laser parameters (such as wavelengths, pulsed widths, laser fluences, heating time, and repetition rates), the size of products can be precisely controlled via the variation of these factors.

In recent years, PLIC-fabricated QDs have been gradually utilized to modify PSCs [27]. Using pulsed laser-induced size-tunable QDs to overcome the weakness of perovskite layers, the PCE and operational stability of PSCs can be effectively enhanced and the crystallinity and compactness of perovskites can be improved by the smaller-sized QDs [27,28,29,30]. For instance, to solve the low efficiency and high energy loss of CsPbI_3_ PSCs that originated from nonradiative recombination, X. He and Z. Liu et al. prepared functionalized MXene QDs (Ti_3_C_2_F_X_) as interface passivators to enhance the performance of PSCs. In their work, by introducing the Ti_3_C_2_F_X_ QDs into CsPbI_3_ PSCs, three beneficial aspects can be obtained. (1) The *p*-type Ti_3_C_2_F_X_ QDs can tune the energy level of perovskite films and provide an efficient pathway for hole transfer; (2) Ti_3_C_2_F_X_ QDs can effectively passivate the structural defects, enhance the crystallinity and compactness of the CsPbI_3_ perovskite layers, and reduce interfacial nonradiative recombination; and (3) Ti_3_C_2_F_X_ QDs form a barrier layer to prevent water invasion and improve the stability of CsPbI_3_ PSCs. As a result, a near-record efficiency of 20.44% for CsPbI_3_ PSCs with a high open-circuit voltage of 1.22 V is obtained [30]. At present, there are several reviews and articles that have concentrated on QD-based PSCs [31,32,33,34]. Specifically, J. Jean has discussed QDs for the efficiency and operational stability of PSCs, and a modified cation exchange method to improve the optoelectronic quality of perovskite nanocrystals was proposed [31]. Y. Zhao et al. have summarized the recent development in integrating semiconductor nanocrystals, including metal chalcogenide QDs, onto bulk perovskite thin films for solar cell device fabrication [32]. Q. Hu, T. Russell, and R. Zhu et al. have investigated the incorporation of zero-dimensional perovskite QDs into three-dimensional perovskite films, which revealed that the modification can heal the surface imperfections in perovskite films, and this is very beneficial for the charge carrier kinetics between the perovskite films and the charge extraction layers [33]. By mixing up-conversion nanoparticles (UCNPs) and carbon QDs in an ideal ratio, M. Alkahtani and M. Abduljawad et al. have developed a promising material to modify the perovskite layer and improve the operational stability and PCE of PSCs, obtaining a PCE value of 20.44% [34]. Different from the previous reports, in this review, we produce an overview and deep introduction of the PLIC method for the preparation of QDs and highlight the innovative works on PLIC-prepared QDs for PSCs. PLIC-prepared QDs with tunable size and PL properties, accompanied by the rapid procedure, have great potential to passivate the structural defects of perovskite layers. In addition, this review not only highlights the advantages of PLIC for the preparation of QDs but also critically points out the challenges and prospects of QD-based PSCs. We believe these discussions will provide clues for the further development of QDs and thus the advancement of QD-based solar cells.

## 2. QD Preparation by PLIC and Optimization for PSCs

### 2.1. Three Types of PLIC from Different Laser Heating Methods

**Figure 2:**
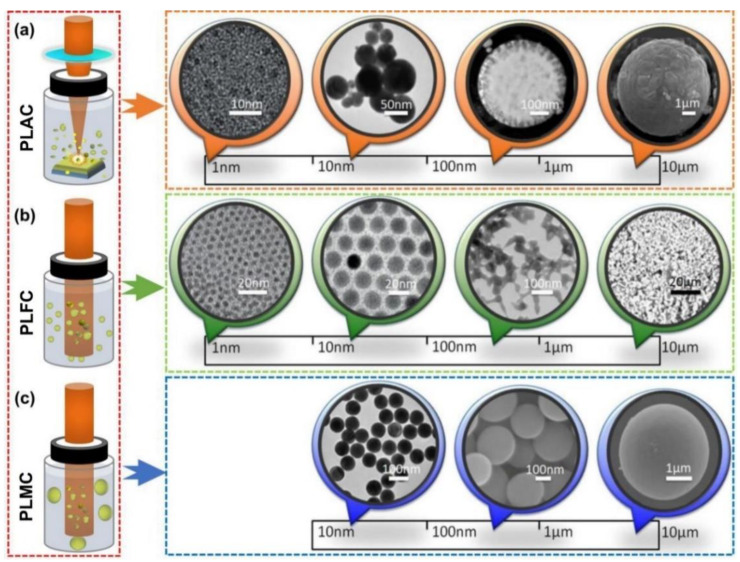
Three types of PLIC. (**a**) The schematic diagram of pulsed laser ablation in colloids (PLAC). (**b**) Pulsed laser fragmentation in colloids (PLFC). (**c**) Pulsed laser melting in colloids (PLFC). Reprinted with permission from Ref. [39].

Nowadays, with the rapid development of laser technology, the pulsed laser with a higher power has been utilized to process various materials in the macroscopic horizon, such as laser cutting [35], laser welding [36], additive manufacturing [37], laser cladding, and laser surface engineering [38], which has promoted industrial development. In micro/nanoscale fields, pulsed laser technologies are also displayed as a powerful tool for the fabrication of nanocrystals and many other nanomaterials, in which PLIC is a typical approach for QD and micro/nanosphere preparation. After uniformly dispersing the precursor materials in liquids (by using ultrasonic oscillation or stirring with magnetic stirrers), the suspension can be rapidly heated by pulsed laser beams assisted by different laser fluences, and the generated plasma of precursor materials can be constrained and quenched by liquids in several nanoseconds to form novel nanomaterials (with different morphologies, sizes, and phases). As shown in Figure 2, according to the different irradiation ways, there are generally three types of PLIC at present [39,40]. 

(1) Pulsed laser ablation in colloids (PLAC). For PLAC (see Figure 2a), a focused laser beam is usually utilized to ablate the bulk materials that are submerged in the liquid medium. The ablated fragments dispersed in the liquids are transformed into colloidal particles, which can be ulteriorly heated by laser energies to form spherical nanoparticles. This method was first proposed by P. Patil et al. in 1987, where an iron targe in aqueous solution was ablated using a 694 nm ruby-pulsed laser, and iron oxides with metastable phases were successfully prepared [41]. Up to now, using this pulsed laser method, a series of nanomaterials, such as carbon QDs, Au nanospheres, core-shell Si@Au sub-micrometer spheres, and AgGe micrometer spheres have been fabricated (size distribution: 1 nm~10 μm) [42]. However, because the focused laser in liquids is in a gradient form, the laser fluence is gradient growth across the incident direction, so this method is only suitable for the preliminary fabrication of nanoparticles with a wide size distribution. In this way, the size distribution of prepared nanomaterials can be hard to control because the gradient form of laser fluences is not uniform, and the focused laser energy ablated on the surface of targets will induce massive numbers of plasma species that cannot be uniformly heated by laser energy in colloids.

(2) Pulsed laser fragmentation in colloids (PLFC). Similarly, PLFC is derived from PLAC (see Figure 2b). During PLFC, the precursor particles are dispersed into the liquids to form colloids, and a high laser fluence beam is directly heated in the colloids (usually excited under an ultraviolet or high-power laser beam). Under the laser heating effect, the initial particles with a bigger average size can be transformed into smaller nanoparticles. This method was proposed by P. Kamat et al. in 1998 [43] and further developed by S. Hashimoto et al. [44]. Their representative work decreased silver colloids with 40–60 nm particle diameters to smaller clusters of 5–20 nm under 355 nm pulsed laser excitation in silver nitrate aqueous solution, and the size reduction originated from effective laser energy absorption in the visible–near-infrared region, where the precise choice of excitation wavelength provided size selectivity in the fragmentation of the clusters [43]. Using this method, some spherical nanoparticles, such as lead sulfide (PbS) QDs, Cd nanospheres, Al nanospheres, and K_2_SiF_6_: Mn^4+^ phosphor particles were fabricated (size distribution: 1 nm~10 μm) [44]. In this way, the size distribution of prepared nanomaterials can be well-controlled. Because the laser beam is unfocused, the colloidal particles dispersed in the liquid medium can be uniformly heated under the high laser fluence, and the smaller products can be easily separated by the centrifugation of liquids.

(3) Pulsed laser melting in colloids (PLMC). Different from the PLAC and PLFC with high-density laser beams, the energy density of PLMC is rather modest. An unfocused laser beam is usually utilized to irradiate the colloids in liquids, and the particle size can be easily tuned by the laser fluences (see Figure 2c). This method was initially proposed by N. Koshizaki et al. in 2010 [45], in which raw CuO nanoparticles with an average size of 34 nm dispersed in acetone were transformed into 300 nm spheres under unfocused laser beam irradiation (laser wavelength: 355 nm, laser fluence: 66 mJ/pulse·cm^2^, 30 min). In recent years, PLMC has been widely used in particle modification. Smaller particles can be melted and reproduced with bigger sizes, such as in the grades of 10 nm~10 μm. The three types of PLIC are closely related to the laser’s interaction with colloids and the size of products is mainly determined by the laser fluences, which can be precisely adjusted via the shape of the laser beam and the laser output power. In this way, similar to PLFC, the size distribution of prepared nanomaterials can also be well-controlled. Because the laser beam is unfocused, the colloidal particles dispersed in the liquid medium can be uniformly heated under the modest laser fluence, producing nanospheres with a bigger size.

### 2.2. PLIC for Preparation of QDs

**Figure 3:**
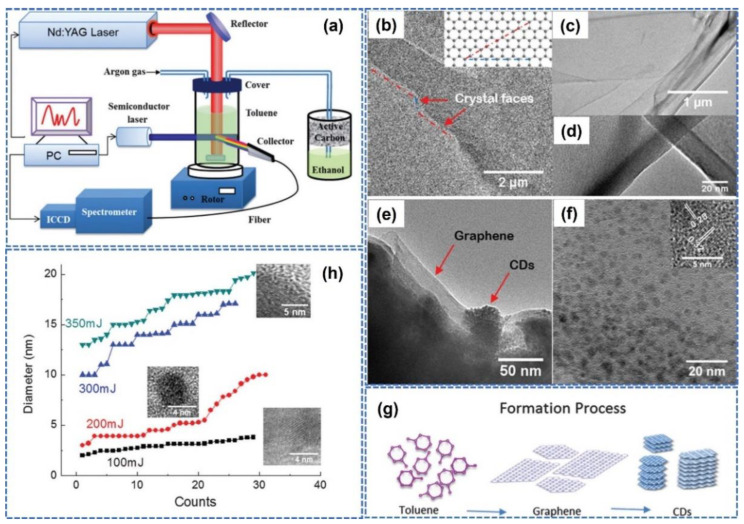
PLIC for carbon QDs preparation. (**a**) Schematic diagram of experimental setup. (**b**–**f**) TEM images of produced carbon QDs under different magnifications are illustrated. (**g**) The schematic diagram for the formation process of carbon QDs. (**h**) The size evolution of carbon QDs under different laser fluences (such as 100, 200, 300, and 350 mJ/pulse). Reprinted with permission from Ref. [26].

In Figure 3, a typical schematic diagram of PLIC for the preparation of QDs is illustrated [26]. In Figure 3a, 5 mL toluene solvent is put into a reaction cell for laser irradiation (the reaction cell is supported by a magnetic stirrer). An unfocused Nd: YAG pulsed laser beam (Quantel brilliant, repetition rate: 10 Hz, pulse width: 8 ns, beam diameter: 8 mm) with a wavelength of 1064 nm is utilized to irradiate the toluene solvent through a quartz window. To protect the safety of the system and environment, argon gas is used to maintain a stable air pressure, and active carbon is utilized to absorb the superfluous gas. During pulsed laser irradiation, a magnetic rotor in toluene solvent is used to keep the solution homogeneous, assisted by the magnetic stirrer. In addition, photoluminescence (PL) is a remarkable property of QDs. To detect the production of carbon QDs in toluene, a real-time PL monitor system is constructed using continuous semiconductor laser stimulation (working wavelength: 450 nm). An optical spectrum instrument coupled with an intensified charge-coupled device (ICCD, Andor Tech., Mechelle 5000) is also used for collecting the PL emission spectrum. In Figure 3b–f, the TEM images of produced carbon QDs under different magnifications are illustrated. The graphene and QDs can be found in the products under the high-resolution transmission electron microscopy (HR-TEM) observation, and the smaller crystal lattice can be measured. In Figure 3g, the schematic diagram for the formation process of carbon QDs is presented, the toluene solvent under heating of the laser energy can be transformed into graphene, and the intermediate products of graphene can be restored under the high temperature to produce carbon QDs. In Figure 3h, the size evolution of carbon QDs under different laser energies (100, 200, 300, and 350 mJ) is investigated. With increasing laser fluences, the average size of carbon QDs is gradually increased, which reveals that the sizes can be controlled below 350 mJ/pulse because the increasing particle size is critically related to lower laser energy (as illustrated in the PLMC model). 

### 2.3. The General Role of Colloidal QDs in PSC Optimization

At present, from their geometrical structure, most high-performance PSCs (also referred to as conventional PSCs) usually contain an “n-i-p” device structure, in which the *n*-type electron transport layer (ETL) and the *p*-type hole transport layer (HTL) are regarded to be the key medium components for separating photogenerated carries. In particular, the ETL plays a significant role in extracting photogenerated electrons from absorbers, transporting them to the conducting substrates, and preventing holes [46]. Hence, if the structural defects in ETL were eliminated, the transmission rate of photogenerated electrons can be greatly improved. In practice, the colloidal QDs can be utilized to repair the defects in ETL via a spin-coating method or layer-by-layer (LBL) deposition, and even colloidal QDs can also be directly utilized as the ETL. For instance, in 2022, M. Grätzel and D. Kim et al. reported a conformal QDs-SnO_2_ ETL for efficient PSCs, in which a compact TiO_2_ blocking layer was covered by a thin layer of polyacrylic acid-stabilized tin oxide QDs (marked as “paa-QD-SnO_2_”) [47]. The uniform bilayer of paa-QD-SnO_2_@TiO_2_ largely improved the perovskite’s absorption of sunlight and formed an outstanding electron-selective contact with the perovskite film. The quantum size effect increased the bandgap of the QDs-SnO_2_ from 3.6 eV to nearly 4.0 eV and produced a corresponding upward shift in its conduction band edge energy. This shift aligned it well with the conduction band edge of the perovskite so that electron capture by the SnO_2_-based ETL proceeded with minimal energy losses. On the contrary, for the inverted PSCs (defined as a “p-i-n” structure), the HTL with lower fill factors is regarded as the charge carrier’s route, the QDs can also be utilized to optimize the charge extraction and minimize interfacial recombination losses of the HTL via reasonably high hole mobility and suitable energy level positions, and the PCE of inverted PSCs can be dramatically improved by enhancing the light-harvesting efficiency. 

**Figure 4:**
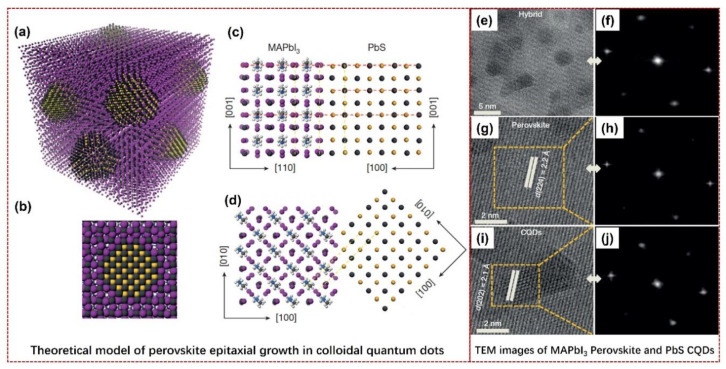
Theoretical model and TEM images of perovskite epitaxial growth in colloidal QDs. (**a**) Three-dimensional atomistic model of QDs in a perovskite matrix. (**b**) A two-dimensional cross-section of a single QD in perovskite. (**c**,**d**) Modeling of PbS QDs and MAPbI_3_ perovskite crystal structures and the interfaces, showing that the perovskite film matches well with PbS in both the X–Z plane and X–Y plane, respectively. (**e**,**f**) TEM and FFT images of the hybrid PbS QDs and MAPbI_3_ perovskite, respectively. (**g**,**h**) TEM and FFT images of the individual MAPbI_3_ perovskite, respectively. (**i**,**j**) TEM and FFT images of the individual PbS QDs, respectively. Reprinted with permission from Ref. [48].

Initially, the pioneering work of colloidal QDs for boosting the PCE of PSCs was demonstrated by E. H. Sargent’s group in 2015 [48], in which the colloidal QDs were utilized to compound with the perovskite materials. The perovskite epitaxial was grown on colloidal QDs and formed a heterojunction. The radiative recombination in QDs can be enhanced by this heteroepitaxy effect, the quantum size effect can be eliminated from the defects of perovskite crystal lattices, and then the electrons/holes in the large-bandgap perovskites can be transferred with a higher efficiency of 80% to become excitons in the QD nanocrystals, which exploits the excellent photocarrier diffusion of perovskite to promote the PCE of perovskite materials. As shown in Figure 4, this theoretical model of perovskite epitaxial growth on colloidal QDs and the real TEM images are presented [48]. Specifically, Figure 4a illustrates a schematic diagram of a three-dimensional atomistic model of QDs in a perovskite matrix. The QDs are sandwiched in the perovskite layers (see Figure 4b, viewed from a two-dimensional cross-section of a single QD in perovskite). In their practical case, a lead sulfide (PbS) QD was utilized in the methylammonium lead iodide (MAPbI_3_) perovskite layers, and the crystal structures of PbS can be well-matched with MAPbI_3_ layers. The interfaces between PbS QD and MAPbI_3_ perovskite were illustrated (see Figure 4c, from the X–Z plane; and Figure 4d, from the X–Y plane). In Figure 4e–j, the TEM and fast Fourier transform (FFT) images of hybrid MAPbI_3_ perovskite and PbS QDs, as well as their states, are also presented, which confirms that the two components of MAPbI_3_ perovskite and PbS QDs can be well-compounded. This heteroepitaxy effect also provides a possible example for improving the PCE of PSCs.

## 3. Pulsed Laser-Prepared QDs for PSCs

To promote the PCE and stability of PSCs, many efforts have been made, including the introduction of new nanomaterials, ligands engineering, ion engineering, and designing different layer structures, such as the coupling effect of colloidal QDs and the construction of stable inverted PSCs [49,50,51]. Herein, using QDs to modify the structural defects of the perovskite layer is one of the most effective ways, as charge carrier transport in the perovskite layer can be promoted and controlled by the special quantum effect of QDs. In recent years, PLIC has been utilized to fabricate various QDs. The precursors mixed in the colloids can be heated by the pulsed laser passivation, and the size distribution of products can be precisely tuned by the laser fluences and other parameters [52,53]. Hence, pulsed laser-prepared QDs have been naturally utilized to modify and boost the PCE value and stability of PSCs. 

### 3.1. Pulsed Laser-Prepared Liquid Metal QDs for PSCs

Hybrid organic–inorganic perovskite materials have attracted extensive attention in the past decades, and the organic lead trihalide MAPbX_3_ (MA = CH_3_NH_3_, X = I, Br, or Cl) is considered a promising material due to its tunable bandgap, long carrier lifetime, high absorption efficiency, and diffusion length. These properties allow MAPbX_3_ materials to be widely used in PSCs [54]. However, for smaller perovskite single crystals at the micro/nanoscale, to match the physical gap between electrodes and perovskite layers that originated from the irregular shape in PSCs, the size of electrodes should be reduced and tight contact must be guaranteed. The desirable liquid metal QDs prepared by PLIC with uniform and size-controllable characteristics (by using ultrasonic oscillation and tunable laser parameters) can be utilized to fill with the defects in ETL, HTL, or perovskite interfaces via a soft contact heteroepitaxy strategy, and the liquid metal QDs can be facilely spin coated or evaporated on the perovskite layer surfaces as electrodes.

**Figure 5:**
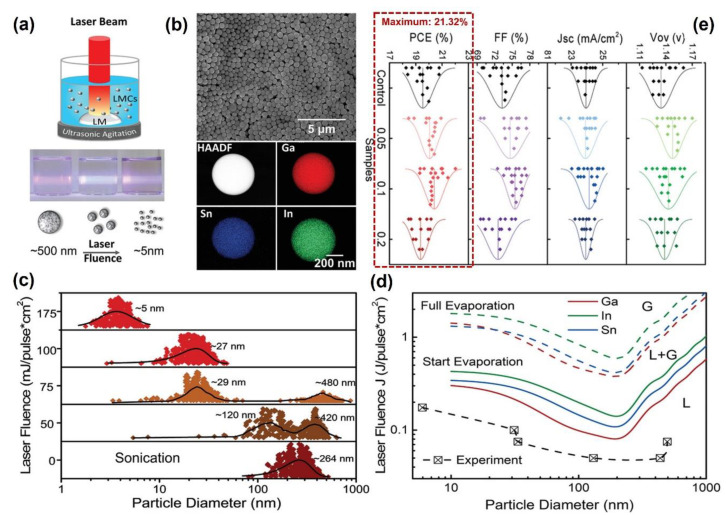
PLIC for preparation of liquid metal alloy QDs and their applications in PSCs. (**a**) Schematic diagram of pulsed laser irradiation in liquids. (**b**) SEM and HAADF images of liquid metal alloys (Ga, In, and Sn compounds). (**c**) Particle size tuning under different laser fluences. (**d**) The phase shifting mechanism of liquid metal alloys (Ga, In, and Sn compounds) under different laser fluences. (**e**) Photovoltaic metrics of devices plotted as functions of liquid metal colloid concentrations. Reprinted with permission from Ref. [55].

Inspired by this heteroepitaxy strategy of compounding perovskite and QDs, in 2020, Y. Du and H. Wang et al. reported a Galinstan QD of supranano liquid metal (an eutectic alloy of Ga, In, and Sn) by laser irradiation in liquids, and the liquid metal QDs served as the defect-repairing medium, displaying a high efficiency in PSCs [55]. In Figure 5a, a schematic diagram of pulsed laser irradiation for liquid metal QD preparation is presented. The raw suspension of liquid metal colloids (LMCs) was dispersed in a glass cell and then uniformly heated under a non-focused pulsed laser beam assisted by an ultrasonic agitation instrument. Figure 5b illustrates a scanning electron microscopy (SEM) image and the corresponding elemental mapping results of Ga, In, and Sn under a high-angle annular dark field model (HAADF, laser fluence: 75 mJ/pulse·cm^2^), which revealed that the components of a Galinstan QD were uniformly distributed. In Figure 5c, LMCs with different sizes can be formed by tuning the laser fluences, and the 5 nm LMCs can be formed at the laser fluence of 175 mJ/pulse·cm^2^. The size-tunning principle was introduced in previous content (see PLFC and PLMC in Section 2). In Figure 5d, the phase-shifting mechanism of the liquid (marked with “L”) and gas (marked with “G”) is illustrated by calculating the thermodynamic function of the “Heating-Melting-Evaporation” (HME) model [56,57,58]. This model was first proposed by the A. Takami group in 1999 and further developed by A. Pyatenko’s group [24,56,57]. The specific mechanism was described in detail in Ref. [57]. Ga, In, and Sn with different sizes can be achieved under different temperatures, and temperature tunning can be realized by pulsed laser fluences. Using this method, not only can the QDs of Ga, In, and Sn alloys be rapidly and effectively prepared but the QDs utilized for repairing the defects of perovskite films can also be obtained, and a peak stable PCE output of 21.32% for PSCs can be achieved (see Figure 5e).

**Figure 6:**
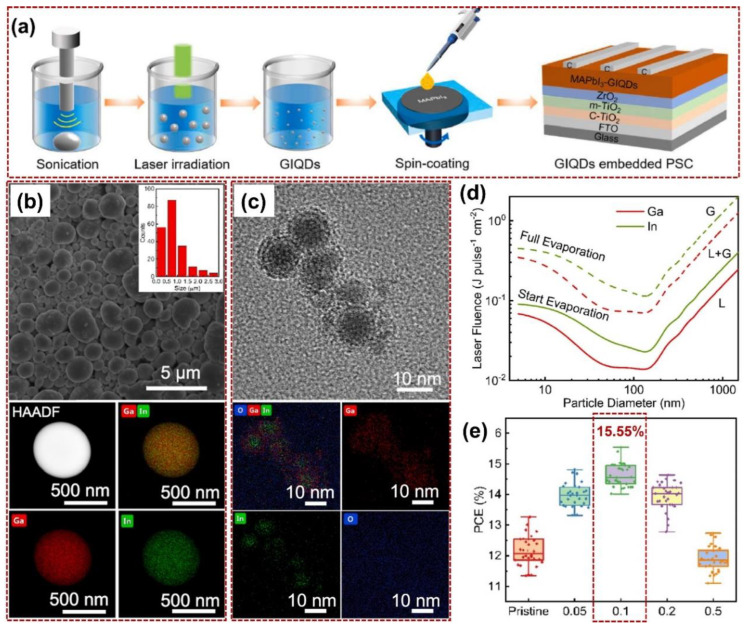
PLIC for preparation of the eutectic gallium–indium (Ga, In alloy) QDs (GIQDs) and their applications in PSCs. (**a**) Schematic diagram of pulsed laser irradiation in liquids for GIQD preparation and construction of the perovskite layer. (**b**) SEM and HAADF images of the pristine liquid metal alloys (Ga, In compounds). (**c**) SEM and HAADF images of the pulsed laser-prepared GIQDs (Ga, In compounds, laser fluence: 100 mJ/pulse·cm^2^). (**d**) The phase-shifting mechanism of the liquid (marked with “L”) and gas (marked with “G”) for gallium–indium. (**e**) Comparison of the PCE of PSCs under different GIQD concentrations (pristine, 0.05, 0.1, 0.2, and 0.5 mg/mL). Reprinted with permission from Ref. [59].

As shown in Figure 6, Y. Yang and X. Li et al. prepared eutectic gallium–indium (Ga, In alloy) liquid metal QDs (marked as “GIQDs”) with a core/shell structure by PLIC, and the prepared GIQDs were utilized to construct the MAPbI_3_ (MA = CH_3_NH_3_) perovskite layer in PSCs [59]. In Figure 6a, during the preparation of GIQDs, to uniformly heat the suspension and prevent the aggregation of liquid metals, the raw suspension of liquid metal colloids was first sonicated using a centrifuge tube (working power: 60 W), and then the post-treatment suspension was irradiated by a non-focused pulsed laser beam (Quantel brilliant B, wavelength: 1064 nm, repetition rate: 10 Hz, pulsed width: 10 ns, beam diameter: 9 mm, irradiation time: 5 min). After preparation, the desired GIQDs with a concentration of 0.1 mg/mL were utilized to construct the perovskite layer using the spin-coating method. Figure 6b,c illustrates the TEM (HAADF) images of the pristine liquid metal alloy and the laser-prepared GIQDs (laser fluence: 100 mJ/pulse·cm^2^). The size of the products can be viewed from the scale bar (pristine: 0.5–1 μm, GIQDs: within 10 nm). Figure 6d illustrates the phase-shifting mechanism of the liquid (marked with “L”) and gas (marked with “G”) for gallium–indium (Ga, In alloy), which is also calculated from the “HME model”. In Figure 6e, the PCE of PSCs under different GIQD concentrations is compared, in which a peak PCE value of 15.55% under 0.1 mg/mL can be obtained.

Herein, the innovative works of pulsed laser-fabricated liquid metal QDs for optimization of the PCE of PSCs was briefly highlighted. From these cases, there are two key issues that should be noticed and need to be overcome in real situations. (1) The aggregation property of liquid metal. As a novel material, the charge carrier’s conduction abilities in liquid metals are rather excellent, and the desirable compact property can be obtained via soft contact, but the aggregation of liquid metals also blocks the uniform preparation of QDs, and thus the electronic property is also restricted. To obtain a uniform liquid metal QD with high quality, a proper method of sonication needs to be employed. An ultrasonic needle with a higher power efficiency can be used for breaking the colloidal suspension. (2) The construction methods of PSCs. In general, as the third generation of solar cells, to eliminate the structural defects of the perovskite medium, using colloidal QDs with the heteroepitaxy effect is considered an effective way, but the modification quality of perovskite film still faces a great challenge. Hence, to obtain a high-quality QD-modified perovskite film, a new method should be adopted. Due to liquid medium alloys with lower melting or boiling points, the atomic layer deposition method (ALD) of colloidal QDs may also be utilized to construct the perovskite layers, and the interfaces between colloidal QDs and the perovskite layer will be more compact. In addition, developing new crafts, such as constructing printable PSCs or stable inverted PSCs, is also beneficial for improving the PCE property of PSCs.

### 3.2. Pulsed Laser-Prepared Carbon QDs for PSCs

**Figure 7:**
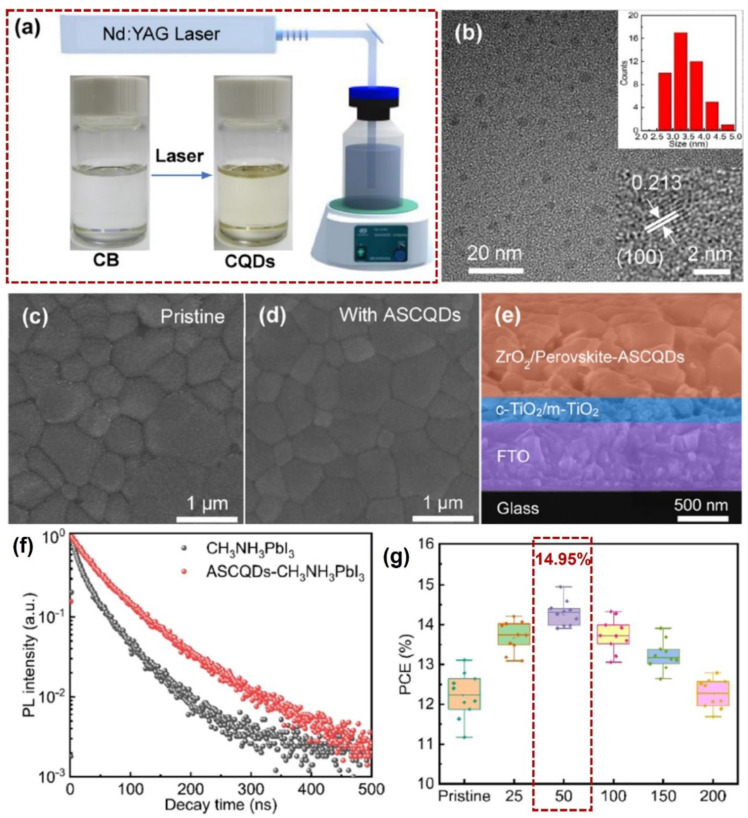
Pulsed laser preparation of ASCQDs for PCE−optimized PSCs. (**a**) Schematic diagram of pulsed laser irradiation in chlorobenzene for ASCQD preparation. (**b**) TEM image and average size distribution of ASCQDs. (**c**,**d**) The SEM images of pristine and ASCQD−modified perovskite films, respectively. (**e**) The cross−section of a multi−layer PSC structure. (**f**) The TR-PL spectrum of a CH_3_NH_3_PbI_3_ perovskite layer and an ASCQD−optimized perovskite layer. (**g**) PCE comparison between different ASCQD−modified PSCs prepared under different laser fluences (pristine, 25, 50, 100, 150, and 200 mJ/pulse·cm^2^). Reprinted with permission from Ref. [60].

The liquid metal QDs utilized as a defect-modification medium display a higher PCE in PSCs, but the price is extensively expensive. To reduce the cost of modification, considering the excellent electrical conductivity of carbon materials, carbon QDs are also used for the optimization of perovskite layers [60,61]. As shown in Figure 7, Y. Yang and X. Li et al. fabricated an anti-solvent carbon QD (ASCQD) by using pulsed laser irradiation in chlorobenzene (CB, see Figure 7a), and the produced ASCQDs (see TEM image in Figure 7b) were utilized as an additive for defect passivation of the grain boundaries of CH_3_NH_3_PbI_3_ in hole-conductor-free, carbon-counter-electrode PSCs [58]. After ASCQD modification, the surface defects of CH_3_NH_3_PbI_3_ perovskite layers can be effectively passivated (see Figure 7c,d). 

Figure 7e illustrates the cross-section of a multi-layer PSC structure, where the ASCQD-optimized perovskite layer serves as the charge carrier transport medium. The carrier extraction and transport performance between the pure CH_3_NH_3_PbI_3_ perovskite layer and the ASCQD-optimized perovskite layer can be analyzed by comparing the intrinsic PL properties. As shown in Figure 7f, the TR-PL spectrum of two components was tested and compared, and the average carrier lifetime of the pristine perovskite film (CH_3_NH_3_PbI_3_, about 41.43 ns) was shorter than that of the ASCQD-optimized perovskite film (ASCQDs-CH_3_NH_3_PbI_3_, about 61.60 ns), confirming that non-radiative recombination was restrained by ASCQD passivation, which was beneficial for achieving a favorable photovoltaic performance. In Figure 7g, a champion efficiency of 14.95% can be achieved under the ASCQDs prepared at 50 mJ/pulse·cm^2^ laser fluence (laser wavelength: 1064 nm, irradiation time: 10 min).

**Figure 8:**
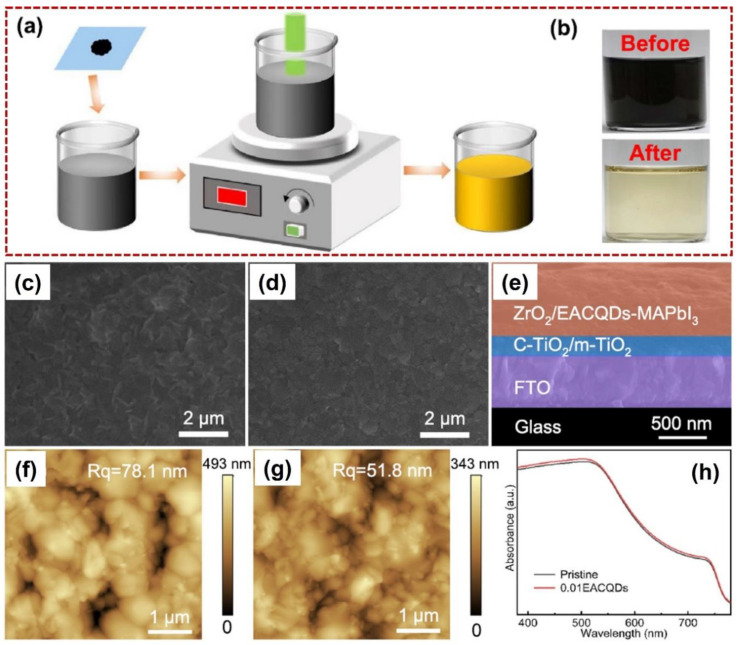
Pulsed laser preparation of EACQDs for PCE-optimized PSCs. (**a**) Schematic diagram of pulsed laser irradiation in carbon nanomaterials diluted in anti-solvent ethyl acetate for EACQD preparation. (**b**) The real images of the carbon colloidal suspension before and after laser irradiation. (**c**,**d**) The SEM images of pristine and EACQD-modified perovskite films, respectively. (**e**) The cross-section of a multi-layer PSC structure. (**f**,**g**) The AFM images of a pristine perovskite film (Rq is 78.1 nm) and an EACQD-optimized perovskite film (Rq is 51.8 nm), respectively. (**h**) The UV–Vis absorbance spectrum of a pristine perovskite film (black line) and an EACQD-optimized perovskite film (red line), confirming the perovskite crystallinity is greatly enhanced after modified with EACQD. Reprinted with permission from Ref. [61].

The same group also fabricated a carbon QD by using pulsed laser non-focused irradiation on a carbon nanomaterial diluted in anti-solvent ethyl acetate (EACQDs) [61]. As shown in Figure 8a, before pulsed laser irradiation, the precursor carbon nanomaterials were firstly dispersed in a glass cell, and then uniformly heated by a laser beam (Quantel brilliant B, laser wavelength: 1064 nm, repetition rate: 10 Hz, pulse width: 10 ns, beam diameter: 9 mm, irradiation time: 10 min). After laser irradiation, the color of the colloidal suspension was transformed to light yellow (see Figure 8b), and the resulting light-yellow solution exhibited a PL phenomenon, depending on the excitation wavelength, which confirmed that the CQDs were successfully prepared. During the PSC device fabrication, the pristine perovskite film with lots of structural defects can be modified by adding a proper concentration of EACQDs (0.01 mg/mL, see SEM images in Figure 8c,d). Figure 8e illustrates the cross-section of EACQD-optimized PSCs (not including the two electrodes). To evaluate the surface quality of pristine and EACQD-modified perovskite films, AFM was utilized to detect surface roughness. After the EACQD modification, surface roughness was decreased from 78.1 nm to 51.8 nm, in accordance with the above SEM images, which was beneficial for the charge carrier’s transportation. In addition, the UV–Vis spectrum of pristine and EACQD-modified perovskite films was also measured. As shown in Figure 8h, the EACQD-modified perovskite film exhibited a slightly higher value than that of the pristine film in the visible light region, which can be ascribed to the enhancement of perovskite crystallinity. As a result, a maximum PCE value of 16.43% can be obtained, which was enhanced by 23.81% when compared with the pristine PSCs, which had a value of 13.27%. Overall, the two innovative works have demonstrated the surface-modified effect of perovskite film via carbon QDs. The introduction of carbon QDs served as an effective ligand for perovskite defect removal. 

### 3.3. Pulsed Laser-Prepared Semiconductor QDs for PSCs

**Figure 9:**
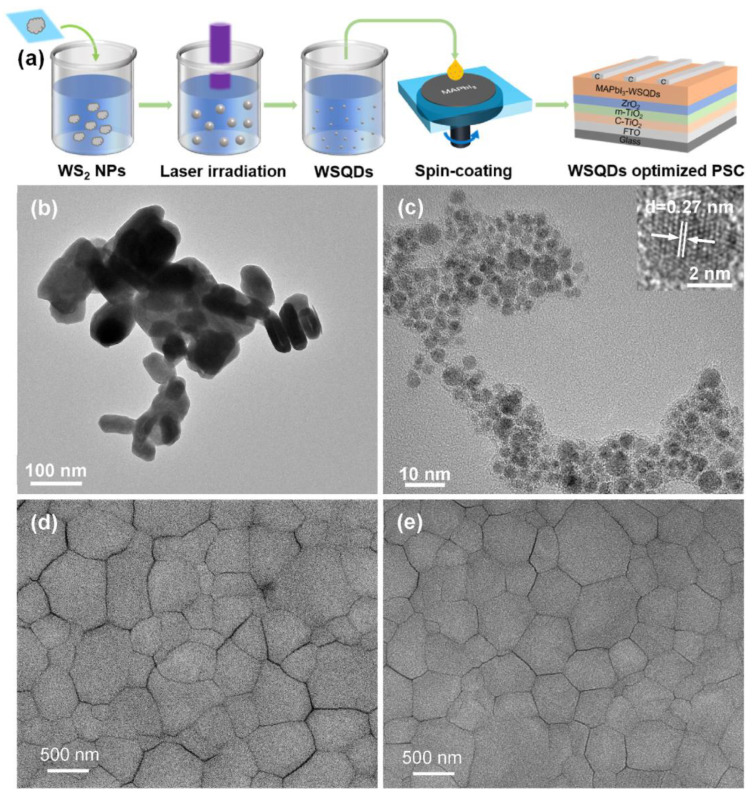
Pulsed laser fabrication of WSQDs for PCE-optimized PSCs. (**a**) Schematic diagram of pulsed laser irradiation in a colloidal WS_2_ suspension in anti-solvent ethyl acetate for preparation of WSQDs, and their utilization for the construction of PSCs. (**b**,**c**) The real TEM images of WS_2_ nanomaterials before and after laser irradiation (particularly, the distance between two arrows is about 0.27 nm, corresponding to the 001 facet of WS_2_), respectively. (**d**,**e**) The SEM images of pristine and WSQD-modified perovskite films, respectively. Reprinted with permission from Ref. [62].

In addition, to decrease the modified cost of perovskite films, many semiconductors and two-dimensional MXene QDs were also utilized to realize cost-effective PSCs [62,63,64]. Thereinto, as a typical sulfide semiconductor material, tungsten sulfide (WS_2_) is an excellent transition metal compound that is widely used in electronic devices. As shown in Figure 9, Y. Yang and X. Li et al. developed a WS_2_ QD using pulsed laser irradiation in anti-solvent ethyl acetate (EA) [62]. 

In Figure 9a, before laser irradiation, the raw WS_2_ nanoparticles (size: 90 nm, purity: 99%) were uniformly dispersed in a glass cell to produce the colloidal suspension, and then the suspension was heated under a non-focused laser beam (Quantel brilliant B, wavelength: 1064 nm, repetition rate: 10 Hz, pulsed width: 10 ns, beam diameter: 9 mm, laser fluence: 600 mJ/pulse·cm^2^) for 5–10 min. After laser irradiation, the WS_2_ QDs (WSQDs) can be obtained, which can be verified by TEM and PL property tests. The prepared WSQDs were utilized as the ligands for MAPbI_3_ perovskite film modification using the spin-coating method, and thus the layered PSCs can be fabricated. As shown in Figure 9b,c, the TEM images of WS_2_ nanoparticles before and after laser irradiation are presented, respectively. The raw WS_2_ with an average particle size of approximately 90 nm was transformed into WSQDs with an average size of 3 nm after laser passivation. In Figure 9d,e, the SEM images of pristine and WSQD-modified perovskite films are illustrated, in which the surface defects of the perovskite film were smoother because the gaps between the perovskite crystals were smaller than the pristine perovskite film, and surface roughness was decreased. As a result, the highest PCE of 16.85% was obtained through WS_2_ QD modification with an optimized concentration of 0.1 mg/mL in the anti-solvent EA, which was substantially promoted when compared with the pristine PSCs (13.27%). In addition, the modified device sample can maintain 71% of the original PCE after 50 days of conversion indoors with a humidity of 30–50%, which demonstrated a novel and fast approach for improving the PCE of PSCs.

**Figure 10:**
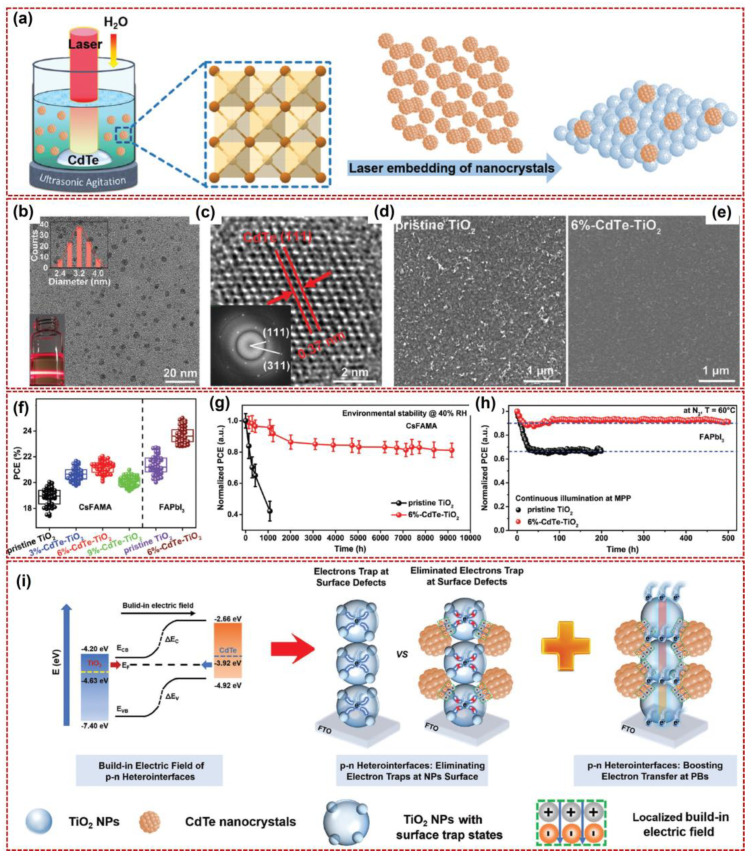
Pulsed laser fabrication of CdTe QDs for PCE−optimized PSCs. (**a**) Schematic diagram of pulsed laser irradiation in the colloidal CdTe suspension for the preparation of CdTe QDs, and their utilization for the embedding of TiO_2_ ETLs in PSCs. (**b**,**c**) The TEM image and HRTEM image corresponding to the FFT of CdTe nanocrystals of pulsed laser−fabricated CdTe nanocrystals (particularly, the distance between two arrows is about 0.37 nm, corresponding to the 111 facet of CdTe), respectively. (**d**,**e**) The SEM images of pristine and CdTe QD−modified TiO_2_ films, respectively. (**f**) PCE distribution of 50 individual CsFAMA−based and FAPbI_3_−based devices. (**g**,**h**) The long−term humidity stability of CsFAMA−based and FAPbI_3_−based devices, respectively. (**i**) Schematic illustration of the energy level shifting of the TiO_2_ ETL before and after being embedded with CdTe nanocrystals to form the *p*-*n* heterointerfaces. Reprinted with permission from Ref. [65].

The innovative works summarized above concentrate on the modification of perovskite layers by laser-produced colloidal QDs to optimize the PCE of PSCs. Developing the ETL with pronounced electron-conducting capability is also very significant. Hence, H. Wang et al. prepared a CdTe nanocrystal using pulsed laser processing in colloids and utilized it for embedding the ETLs of the TiO_2_ layer in PSCs, in which a champion efficiency of 25.05% was obtained [65]. As shown in Figure 10, a schematic diagram of PLIC for CdTe nanocrystal preparation is presented. The size-controlled CdTe nanocrystals can be generated using pulsed laser irradiation in bulk CdTe materials immersed in deionized water (Quantel, laser wavelength: 1064 nm, repetition rate: 10 Hz, pulse width: 8 ns, beam diameter: 8 mm), and then the prepared nanocrystals can be embedded into the TiO_2_ ETL layers by using a chemical bath deposition. The CdTe nanocrystals made using the pulsed laser can be used to decorate the TiO_2_ ETL layers. Figure 10b,c illustrates the TEM image and HR-TEM image corresponding to the FFT of CdTe nanocrystals (lattice spacing: 0.37 nm), with an average particle size distribution of 3 nm, confirming that the CdTe QDs were successfully obtained (with a 6% content of CdTe). After modification of TiO_2_ ETLs by CdTe QDs, the surface of pristine TiO_2_ was transformed to be smoother, and the roughness was also reduced. To optimize the PCE in different PSCs, performance and stability were also tested. As shown in Figure 10f, the PCE values of PSCs for TiO_2_ ETLs modified with different contents of CdTe are provided. For perovskites Cs_0.05_(FA_0.85_MA_0.15_)PbI_2.55_Br_0.45_ (marked as “CsFAMA”) and FAPbI_3_, a higher PCE value of 23.81 ± 0.85% (average best: 25.05%) can be achieved using CdTe QD modification under a concentration of 6%. In Figure 10g,h, the long-term humidity durability of all unencapsulated CsFAMA-based and FAPbI_3_-based devices was examined under 40% relative humidity (RH) in a dark space at room temperature. The results revealed that, compared with the control device (using the pristine TiO_2_ ETLs without CdTe modification), the CdTe-modified CsFAMA and FAPbI_3_ perovskite layers displayed more stable features. For CsFAMA perovskites, almost all maintained 81% of their initial PCE over 9000 h; for FAPbI_3_ perovskite, over 90% of the initial PCE was maintained for 500 h. This enhancement was attributed to the improved crystallinity and the decreased defect states, with the surface of TiO_2_ ETLs being more compact. For multi-layer PSCs, the modification of perovskite or ETL by proper QDs is beneficial for boosting device performance. In Figure 10i, to explain the mechanism for optimization, a schematic illustration of the energy level shift of TiO_2_ ETL before and after being embedded with CdTe nanocrystals is presented. Using pulsed laser irradiation in the liquid to produce well-defined *p*-type CdTe nanocrystals in desired solvents, it is possible to embed laser-generated *p*-type CdTe nanocrystals at the particle boundaries (PBs) of the *n*-type TiO_2_ ETLs, forming *p*-*n* heterointerfaces. The embedded *p*-*n* heterointerfaces can efficiently inhibit the carrier loss at the PBs, owing not only to the elimination of electron trapping at the TiO_2_ nanoparticles but also the boosted electron transfer between adjacent TiO_2_ nanoparticles through a localized built-in electric field. By elaborately modulating the concentration of embedded *p*-type CdTe nanocrystals, the electron mobility of the ETLs can be enhanced from 2.67 × 10^−5^ up to 1.89 × 10^−2^ cm^2^ V^−1^s^−1^. The embedding of CdTe nanocrystals also modulates the crystallization kinetics of the TiO_2_ matrix, which is favorable for the inhibition of rutile TiO_2_ that is detrimental to both electron conduction and the light-induced stability of perovskite. Owing to boosted electron conduction at the ETL and the subsequent suppression of charge accumulation and recombination at the interface between the ETL and the perovskite, a higher efficiency of 25.05% can be obtained.

The innovative works introduced above focus on pulsed laser-prepared QDs for boosting the PCE of PSCs. Compared with the pristine PSCs, after the charge carrier transport layers are modified with QDs, the PCE of PSCs is greatly enhanced. As listed in Table 1, the QDs prepared by PLIC and used in PSCs are generally illustrated, including the different average sizes and the structures of the devices. Here, the QDs prepared by laser-generated CdTe nanocrystals exhibit the highest PCE of 25.05%, which can be attributed to the interface between the ETL and CdTe nanocrystal being more compact, as well as improved crystallinity [65].

## 4. Discussion on the Possible Routes and Prospects for PCE Optimization of PSCs

As an overview, for optimizing the PCE of PSCs, using QD modification was only considered as an effective strategy, and pulsed laser preparation of colloidal QDs just served as a facial approach. PLIC has been proven to be an excellent approach for colloidal QD preparation, in which the size and purity of products can be precisely controlled by tuning the pulsed laser fluences and other parameters. The smaller size of QDs can be utilized to remove the structural defects, and the quantum effect can also be used for tuning the local state of embedded materials and the compact property. This strategy is beneficial for boosting the transport of charge carriers in PSCs [66]. Meanwhile, in terms of other aspects, developing chemical ligand/ion additive agents and constructing novel structures for PSCs also display wonderful performances, which provide many more possible routes for PCE optimization of PSCs [67,68].

### 4.1. The Breakthrough of PCE for Inverted PSC Optimization

Recently, as illustrated in Section 1, the champion PCE of inverted PSCs has achieved a certified 26.54% [3]. Compared with the conventionally structured PSCs (“n-i-p” type), the development of improved self-assembled molecules (SAMs) and the passivation strategy in the inverted PSCs exhibits an interfacial modification for HTL [69,70]. However, the poor wettability and intrinsic agglomerations of SAMs will also cause interfacial losses, impeding further improvement in the PCE and operational stability. To overcome this weakness, a strategy of using a molecular hybrid at the buried interface of inverted PSCs is proposed by Z. Liu, N. Park, and W. Chen et al. [3], in which a co-assembling of multiple carboxylic acid functionalized aromatic compounds of 4, 4′, 4″-nitrilotribenzoic acid (NA) and a popular SAM of [4-(3, 6-dime-thyl-9H-carbazol-9-yl) butyl] phosphonic acid (Me-4PACz) is utilized to improve the interfacial characteristics. Using the compound of SAM/NA to add, on the surface, a nickel oxide (NiO) substrate for preparation of inverted PSCs, the wettability of the perovskite solution on the HTL is improved, leading to the reduction of nanovoids and the release of stress at the buried interface. Due to the interaction between Me-4PACz and the triphenylamine moiety in the NA molecule, the NA/Me–4PACz compound can reduce the agglomeration of Me-4PACz to homogenize the distribution of Me-4PACz, thus facilitating carrier extraction and reducing nonradiative recombination at the NiO/perovskite interface. In this present work, the proper choice of NA is determined by the experimental comparison of the reduced agglomeration effect of Me-4PACz between benzoic acid (BA), trimesic acid (TA), and NA, in which the NA sample shows superiority. Meanwhile, the density functional theory (DFT) calculation also indicated that the absorbed Me-4PACz on the perovskite surface has a preferred configuration for defect passivation, and a π–π interaction between Me-4PACz and the NiO surface leads to much higher absorption energy. This could break the Me-4PACz tetramers and reduce its agglomeration to homogenize its distribution, leading to compact and uniform NiO/Me-4PACz HTL at the buried interface. In terms of these inverted PSCs, modification of HTL using the pulsed laser-fabricated QDs has been rarely reported, as the cross-study of laser micro/nano-processing technology and the field of PSCs is very unique and this investigation is still ongoing (which is also called the “laser embedding strategy”) [7,71]. Therefore, combined with the advantages of PLIC for the preparation of QDs, this strategy will provide new eyesight into future research. Meanwhile, considering the structural defects in perovskite layers, the defect states can be removed and the interface between the perovskite layer and the charge carrier transport layer can be made more compact by using laser-fabricated perovskite QDs to fill in the perovskite layers.

### 4.2. Exchanging Chemistry and Structure Engineering for PSC Optimization

Nowadays, there are also many innovative and popular ways to optimize the PCE of PSCs, the first method is exchanging chemistry, including the chemical ligand/ion engineering and its utilization for structural defect passivation [72,73]. As we all know, embedding chemical ligands/ions in the perovskite materials will improve the degree of perovskite crystallization, and the introduction of chemical ligands/ions is also beneficial for promoting the interface’s compactness and the operational stability between the perovskite layer and the conductive layer, which reduces energy loss during charge extraction [74]. On the other hand, potential issues such as ligand desorption and recombination loss will also balance the optimism around these methods.

For instance, J. You et al. proposed a surface passivation strategy via an organic halide salt phenethyl ammonium iodide (PEAI) and HC(NH_2_)_2_-CH_3_NH_3_ mixed perovskite film. The PEAI embedded on the perovskite film can reduce the defect states and suppress non-radiative recombination, and a certified efficiency of 23.32% was achieved in PSCs [75]. M. Guo et al. developed a ligand passivation method for perovskite film by using amine molecules, in which three amine-based molecules (formamidine iodide, octyl ammonium iodide, and aromatic nicotinic acid) were utilized to passivate the surface defects in perovskite film [76]. In particular, formamidine iodide was found to react preferentially with the PbI_2_ at the grain boundary of perovskite film, promoting secondary grain growth and resulting in a 21% increase in grain size. Because of the reduction in grain boundaries and the combined passivation of MA^+^ and I^−^ vacancies, the PCE of formamidine iodide (FAI)-passivated PSCs increased from 18.63% to 19.35% in ambient air and maintained 91% of their initial values after 76 days of storage under conditions of 20 ± 5 °C and <5% RH. W. Chen, C. Brabec, and Y. Li et al. proposed a pseudo-halogen thiocyanate (SCN^-^) ion that was utilized to enhance crystallization and reduce the grain boundaries of iodide/bromide mixed halide wide-bandgap perovskites (FA_0.8_Cs_0.2_PbI_1.6_Br_1.4_) [77]. The trace amount of SCN^−^ ions in the bulk entered the perovskite lattice, forming an I/Br/SCN^−^ solid solution and occupying the iodine vacancies, which block halide ion migration via steric hindrance. These effects retarded halide phase segregation under operation and reduced energy loss in the wide-bandgap perovskite cells. The resulting perovskite/organic tandem solar cell achieved a certified PCE of 25.06% and obtained an operational stability of 1000 h. These innovative works have indicated that the dopant-addictive synergism is beneficial for boosting the PCE and operational stability of PSCs, and that choosing a proper addictive agent is dependent on the structures and components of the PSCs.

**Figure 11:**
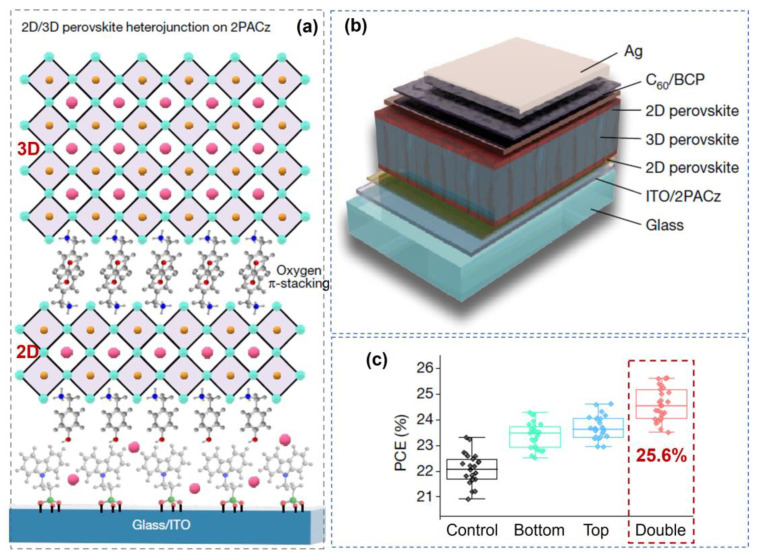
Design of a 2D/3D perovskite heterojunction and test of their PCE in PSCs. (**a**) Schematic diagram of the designed 2D/3D perovskite heterojunction. (**b**) The structural diagram of the designed inverted PSCs. (**c**) Comparison of PCE distribution for PSCs with different structures (control, bottom, up, and double side). Reprinted with permission from Ref. [5].

Another strategy is structure design engineering. To improve the PCE and stability of PSCs, several new structural PSCs were constructed. Except for the conventional “n-i-p” structure, the inverted PSCs served as outstanding delegates. The crucial core of inverted PSCs is the compact interface between the inverted perovskite layer and the HTL, in which the charge carriers generated from the perovskite medium can be rapidly transferred via the sensitized layer of the HTL after harvesting the solar energy [78]. Meanwhile, the dimensionally graded structure with a heterojunction also provides a promising strategy to optimize the energy alignment, improve charge excitation and transport for the perovskite absorber, and thus favor the advancement of device performance [79]. For instance, R. Azmi and S. Wolf et al. designed a double-side 2D/3D heterojunction for inverted PSCs [5]. As shown in Figure 11, a schematic diagram of the designed 2D/3D perovskite heterojunction is illustrated (Figure 11a), in which the 2D and 3D perovskites were fabricated via thermal evaporation, spin-coating ligands, and the annealing approach. The interface defects between 2D top and 3D bottom perovskites were modified using alkyl amine ligands, and the interface can be strengthened via acid-based reactions with the phosphonic acid group from the organic hole-transporting self-assembled monolayer molecule. In Figure 11b, the structural diagram of the designed inverted PSCs is illustrated, and a double-sided 2D/3D heterojunction was formed. Compared with the PSCs with other structures (only one 2D perovskite layer loaded on the top or bottom of 3D), a champion PCE value of 25.6% (certified 25.0%) can be achieved (see Figure 11c). The PSCs also retained 95% of their initial PCE after 1,000 h of one-sun illumination at 85 °C in air. The enhanced performance originated from the coupling effect between 2D and 3D heterojunctions, where the introduction of alkyl amine ligands promoted the interface’s compactness and degree of perovskite crystallization [80].

### 4.3. Superiority Comparison between the Laser-Made and Chemical Approaches for QDs Used in PSC Optimization

The advantage of PLIC for QDs used in PSCs was introduced in Section 1. Compared with chemical approaches, such as the hot injection method, the colloidal microemulsion method, and the sol-gel method, the relative merits of the two strategies can be outlined. (1) The size effect of QDs on the nanoscale can be easily tuned by using pulsed laser heating in colloids, and this is a great challenge and even impossible for chemical methods. Even though the colloidal crystal template method can be utilized for QD preparation via self-assembling, the issue of precisely controlling the size still faces many difficulties. (2) Special structures, such as QDs with core/shell structures that are required by PSCs to improve their performance, can be prepared via PLIC, assisted by inert gas, and these QDs cannot be rapidly obtained by chemical routes. This is because, during the chemical procedures, QDs with core/shell structures are formed by the precursor materials with different diffusion rates, requiring a long time to migrate between the inner and surface areas; however, this exchange process can be easily achieved by using pulsed laser heating-induced electron injection [55,59]. (3) Compared with the chemical approach, the limited yields of pulsed laser-prepared QDs remain a great weakness due to the fact that the colloids of QD precursors for laser heating are contained in a bottle with a small volume. In a real situation, if massive QD products were required in PSC studies, the pulsed laser methods should be repeated numerous times. However, as for the chemical routes, the initial precursor materials can be sufficiently prepared and many products can be naturally harvested. (4) QDs with neat purity are crucial for the modification of perovskite films in PSCs. For chemical routes, the produced QDs usually contain lots of chemical groups that are not neat, due to a series of complex procedures accompanied by the control of proper reaction conditions. On the other hand, for PLIC, the QDs are generated from the optothermal effect between the laser beam and precursors, meaning that the QDs are directly produced from the precursors without additional chemical agents and reactions. Therefore, neat products can be easily obtained. 

### 4.4. The Industrial Tendency for Commercial PSC Optimization

**Figure 12:**
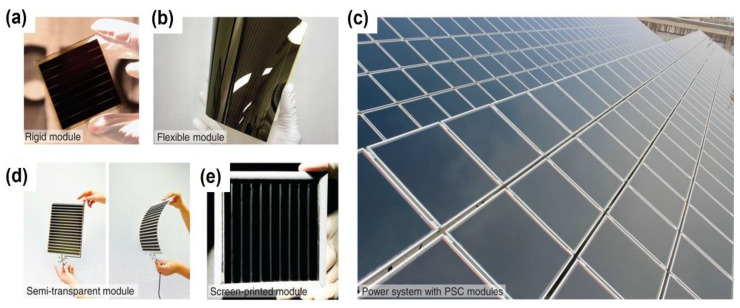
Perovskite solar modules. (**a**) Rigid perovskite mini-module. (**b**) Roll-to-roll processed flexible module. (**c**) Power system with printable triple mesoscopic PSC module. (**d**) Semi-transparent module fabricated via an inkjet printing technique. (**e**) Screen-printed module prepared by Wuhan National Laboratory for Optoelectronics (WNLO) in Huazhong University of Science and Technology (HUST). Reprinted with permission from Ref. [81].

In addition, apart from the aspects above, the tendency of industrial applications also needs to be considered. The terminus purpose of investigations on promoting PCE values and the stability of PSCs is their utilization in real applications. At present, the PSCs are gradually progressing from laboratory research to photovoltaic factories. For instance, S. Seok, M. McGehee, E. Sargent, and H. Han et al. have reviewed the challenges and prospects for commercializing PSCs [81]. To ensure the PCE value and stability of PSCs, a proper perovskite material should be obtained, and the perovskite modules should be easily fabricated from the techniques. As shown in Figure 12, several perovskite modules for commercial PSCs were developed, including the smaller rigid module, the flexible module, the printable module, the semi-transparent module, and the screen-printed module prepared by relevant universities and photovoltaic factories. Industrial products have sped up the commercial advances of PSCs.

## 5. Summary and Outlook

In summary, focusing on the great potential of colloidal QDs embedded in PSCs for the enhancement of sunlight absorbency and the charge carrier transport layer, this review summarized the innovative works of colloidal QD-modified perovskite layers for improving the PCE and operational stability of PSCs. Combined with the unique advantages of PLIC for colloidal QD preparation, pulsed laser-fabricated liquid metal QDs, carbon QDs, and semiconductor QDs utilized for optimizing the performance of PSCs were briefly introduced. Regarding the advantages of PLIC for QD preparation, QDs with a desirable size can be easily prepared by PLIC, and the particle size-controlled mechanism is closely related to the laser fluences and other laser parameters. The special structure of core/shell QDs can be easily prepared by PLIC in conjunction with inert gas. The neat purification of QDs can be achieved by direct pulsed laser irradiation in colloids, without the introduction of other chemical agents. In addition, to optimize the defect states of perovskite layers for charge carrier transport and ensure the interface’s compactness between the perovskite medium and colloidal QDs, a proper technique should be developed. At present, the atomic layer deposition method (ALD) has been widely utilized for the construction of functional films and may display great value in this field because colloidal QDs with smaller sizes on the nanoscale can be easily evaporated and deposited in the ALD instrument. In addition, regarding challenges and prospects, for QDs made using PLIC, existing problems mainly include small production yields due to the restriction of smaller bottles (typically mg/bottle). However, with the development of advanced pulsed laser manufacturing, pulsed lasers with higher power have been utilized in industrial production, so that larger vessels and laser beam spot sizes can be utilized to fabricate the materials at the micro/nanoscale, and the yields of production will be massively improved. Meanwhile, according to the different structures of PSCs, finding the proper chemical ligands/ions to promote perovskite layer crystallization and compactness is also very significant. Many suitable methods for ligand/ion engineering in previous reports have been utilized for the surface passivation of perovskite films. In particular, the mixture of QDs and ligands/ions will be a reliable strategy for the optimization of ETL/HTL, as the synergistic effect between QDs and ligands/ions can be displayed. Furthermore, designing novel structured PSCs is also a reliable strategy for PSC optimization, such as the construction of inverted PSCs and dimensionally graded structures with a 2D/3D heterojunction. For industrial applications, a stable and higher-efficiency PSC product should be produced from real technology. At present, several perovskite modules for commercial PSCs have been developed, and these efforts are beneficial for creating a cost-effective and environmentally friendly future. Overall, this review not only highlighted the innovative works of pulsed laser-prepared colloidal QDs for the optimization of PSCs but also pointed out the practical issues that need to be considered, providing great benefits for future investigations in this field (such as perovskite solar cells, nanomaterials, and other functional devices).

## Figures and Tables

**Table 1:** The QDs used in PSCs prepared using PLIC.

QDs	Size	Structure of Devices	Laser Types	Modified Layers	PCE	References
Ga, In, and Sn	5 nm	QD/perovskite/TiO_2_	Quantel	ETL	21.32%	[55]
Ga, In	5.5 nm	GIQD/ZrO_2_/TiO_2_	Quantel	ETL	15.55%	[59]
ASCQD	3.2 nm	ASCQD/perovskite	Quantel	Perovskite layer	14.95%	[60]
EACQD	5.37 nm	EACQD/ZrO_2_/TiO_2_	Quantel	Interfaces	16.43%	[61]
WSQD	3 nm	WSQD/ZrO_2_/TiO_2_	Quantel	ETL	16.85%	[62]
CdTe	3.2 nm	CdTe/TiO_2_	Quantel	ETL	25.05%	[65]

## Data Availability

Not applicable.

## References

[1] Singh H., Dey P., Chatterjee S., Sen P., Maiti T. (2021). Formamidinum containing tetra cation organic-inorganic hybrid perovskite solar cell. Sol. Energy.

[2] Cheng P., An Y., Jen A., Lei D. (2024). New nanophotonics approaches for enhancing the efficiency and stability of perovskite solar cells. Adv. Mater..

[3] Liu S., Li J., Xiao W., Chen R., Sun Z., Zhang Y., Lei X., Hu S., Kober-Czerny M., Wang J. (2024). Buried interface molecular hybrid for inverted perovskite solar cells. Nature.

[4] Zhu P., Wang D., Zhang Y., Liang Z., Li J., Zeng J., Zhang J., Xu Y., Wu S., Liu Z. (2024). Aqueous synthesis of perovskite precursors for highly efficient perovskite solar cells. Science.

[5] Azmi R., Utomo D.S., Vishal B., Zhumagali S., Dally P., Risqi A.M., Prasetio A., Ugur E., Cao F., Imran I.F. (2024). Double-side 2-dimensional/3-dimensional heterojunctions for inverted perovskite solar cells. Nature.

[6] Wang P., Li R., Chen B., Hou F., Zhang J., Zhao Y., Zhang X. (2020). Gradient energy alignment engineering for planar perovskite solar cells with efficiency over 23%. Adv. Mater..

[7] Yang X., Zhao W., Li M., Ye L., Guo P., Xu Y., Guo H., Yu H., Ye Q., Wang H. (2022). Grain-boundaries-engineering via laser manufactured La-Doped BaSnO_3_ nanocrystals with tailored surface states enabling perovskite solar cells with efficiency of 23.74%. Adv. Funct. Mater..

[8] Pelicano C., Yanagi H. (2018). Effect of rubrene: P3HT bilayer on photovoltaic performance of perovskite solar cells with electrodeposited ZnO nanorods. J. Energy Chem..

[9] Chen M., Wang Y., Zhang Y. (2019). Enhanced light absorption of textured perovskite solar cells employing two-dimensional nanoarrays. J. Photon. Energy.

[10] Chi W., Banerjee S. (2022). Application of perovskite quantum dots as an absorber in perovskite solar cells. Angew. Chem. Int. Edit..

[11] Chen H., Pina J., Hou Y. (2021). Sargent. Synthesis, applications, and prospects of quantum-dot-in-perovskite solids. Adv. Energy Mater..

[12] Jin X., Zhang X., Fang H., Deng W., Xu X., Jie J., Zhang X. (2018). Facile Assembly of high-quality organic-inorganic hybrid perovskite quantum dot thin films for bright light-emitting diodes. Adv. Funct. Mater..

[13] Taghipour N., Dalmases M., Whitworth G., Dosil M., Othonos A., Christodoulou S., Liga S., Konstantatos G. (2023). Colloidal quantum dot infrared lasers featuring sub-single-exciton threshold and very high gain. Adv. Mater..

[14] Choi M., Lee M., Park S., Kim B., Jun S., Park S., Song J., Ko Y., Cho Y. (2023). Single quantum dot selection and tailor-made photonic device integration using a nanoscale-focus pinspot. Adv. Mater..

[15] https://www.nobelprize.org/prizes/chemistry/.

[16] Jang E., Jang H. (2023). Review: Quantum dot light-emitting diodes. Chem. Rev..

[17] Song J., Wang O., Shen H., Lin Q., Li Z., Wang L., Zhang X., Li L. (2019). Over 30% external quantum efficiency light-emitting diodes by engineering quantum dot-assisted energy level match for hole transport layer. Adv. Funct. Mater..

[18] Kim T., Kim K., Kim S., Choi S., Jang H., Seo H., Lee H., Chung D., Jang E. (2020). Efficient and stable blue quantum dot light-emitting diode. Nature.

[19] Li H., Peng Q., Xu X., Wang J. (2023). Quantum dots for optoelectronics. Adv. Photonics.

[20] Yao Y., Yang Z., Hwang J., Su H., Haung J., Lin T., Shen J., Lee M., Tsai M., Lee Y. (2017). Coherent and polarized random laser emissions from colloidal CdSe/ZnS quantum dots plasmonically coupled to ellipsoidal Ag nanoparticles. Adv. Opt. Mater..

[21] Enomoto K., Inoue D., Pu Y. (2019). Controllable 1D patterned assembly of colloidal quantum dots on PbSO_4_ nanoribbons. Adv. Funct. Mater..

[22] Swarnkar A., Chulliyil R., Ravi V., Irfanullah M., Chowdhury A., Nag A. (2015). Colloidal CsPbBr_3_ perovskite nanocrystals: Luminescence beyond traditional quantum dots. Angew. Chem. Int. Edit..

[23] Li Y., Zheng Z., Yan J., Lu B., Li X. (2022). A review on pulsed laser preparation of nanocomposites in liquids and their applications in photocatalysis. Catalysts.

[24] Pyatenko A., Wang H., Koshizaki N. (2014). Growth mechanism of monodisperse spherical particles under nanosecond pulsed laser irradiation. J. Phys. Chem. C..

[25] Li X., Wang H., Shimizu Y., Pyatenko A., Kawaguchi K., Koshizaki N. (2011). Preparation of carbon quantum dots with tunable photoluminescence by rapid laser passivation in ordinary organic solvents. Chem. Commun..

[26] Yu H., Li X., Zeng X., Lu Y. (2016). Preparation of carbon dots by non-focusing pulsed laser irradiation in toluene. Chem. Commun..

[27] Afta S., Iqbal M., Hussain S., Kabir F., Al-Kahtani A., Hegazy H. (2023). Quantum junction solar cells: Development and prospects. Adv. Funct. Mater..

[28] Ahmad W., He J., Liu Z., Xu K., Chen Z., Yang X., Li D., Xia Y., Zhang J., Chen C. (2019). Lead selenide (PbSe) colloidal quantum dot solar cells with >10% efficiency. Adv. Mater..

[29] Chi W., Banerjee S. (2022). Performance improvement of perovskite solar cells by interactions between nano-sized quantum dots and perovskite. Adv. Funct. Mater..

[30] Xu D., Li T., Han Y., He X., Yang S., Che Y., Xu J., Zou H., Guo X., Wang J. (2022). Fluorine functionalized MXene QDs for near-record-efficiency CsPbI3 solar cell with high open-circuit voltage. Adv. Funct. Mater..

[31] Jean J. (2020). Getting high with quantum dot solar cells. Nat. Energy.

[32] Chen Y., Zhao Y. (2020). Incorporating quantum dots for high efficiency and stable perovskite photovoltaics. J. Mater. Chem. A.

[33] Yang W., Su R., Luo D., Hu Q., Zhang F., Xu Z., Wang Z., Tang J., Lv Z., Yang X. (2020). Surface modification induced by perovskite quantum dots for triple-cation perovskite solar cells. Nano Energy.

[34] Alkahtani M., Alenzi S., Alsolami A., Alsofyani N., Alfahd A., Alzahrani Y., Aljuwayr A., Abduljawad M. (2022). High-performance and stable perovskite solar cells using carbon quantum dots and upconversion nanoparticles. Int. J. Mol. Sci..

[35] Liu Y., Zhang S. (2024). Modeling of separation speed in thick plate cutting with a high-power fiber laser. Opt. Laser Technol..

[36] Lei Z., Cao H., Cui X., Ma Y., Li L., Zhang Q. (2024). A novel high efficiency narrow-gap laser welding technology of 120 mm high-strength steel. Opt. Laser Eng..

[37] Sun H., Zou B., Wang X., Chen W., Zhang G., Quan T., Huang C. (2024). Advancements in multi-material additive manufacturing of advanced ceramics: A review of strategies, techniques and equipment. Mater. Chem. Phys..

[38] Wu Q., Long W., Zhang L., Zhao H. (2024). A review on ceramic coatings prepared by laser cladding technology. Opt. Laser Technol..

[39] Zhang D., Gökce B., Barcikowski S. (2017). Laser synthesis and processing of colloids: Fundamentals and applications. Chem. Rev..

[40] Li Y., Xiao L., Zheng Z., Yan J., Sun L., Huang Z., Li X. (2023). A review on pulsed laser fabrication of nanomaterials in liquids for (photo) catalytic degradation of organic pollutants in the water system. Nanomaterials.

[41] Patil P., Phase D., Kulkarni S., Ghaisas S., Kulkarni S., Kanetkar S., Ogale S., Bhide V. (1987). Pulsed-laser-induced reactive quenching at liquid-solid interface: Aqueous oxidation of iron. Phys. Rev. Lett..

[42] Zhang D., Gökce B., Notthoff C., Barcikowski S. (2015). Layered seed growth of AgGe football-like microspheres via precursor-free picosecond laser synthesis in water. Sci. Rep..

[43] Kamat P., Flumiani M., Hartland G. (1998). Picosecond dynamics of silver nanoclusters photoejection of electrons and fragmentation. J. Phys. Chem. B.

[44] Werner D., Hashimoto S. (2013). Controlling the pulsed laser induced size reduction of Au and Ag nanoparticles via changes in the external pressure, laser intensity, and excitation wavelength. Langmuir.

[45] Wang H., Pyatenko A., Kawaguchi K., Li X., Swiatowska-Warkocka Z., Koshizaki N. (2010). Selective pulsed heating for the synthesis of semiconductor and metal submicrometer spheres. Angew. Chem. Int. Ed..

[46] Zhou J., Lyu M., Zhu J., Li G., Li Y., Jin S., Song J., Niu H., Xu J., Zhou R. (2022). SnO_2_ Quantum dot-modified mesoporous TiO_2_ electron transport layer for efficient and stable perovskite solar cells. ACS Appl. Energy Mater..

[47] Kim M., Jeong J., Lu H., Lee T., Eickemeyer F., Liu Y., Choi I., Choi S., Jo Y., Kim H. (2022). Conformal quantum dot-SnO_2_ layers as electron transporters for efficient perovskite solar cells. Science.

[48] Ning Z., Gong X., Comin R., Walter G., Fan F., Voznyy O., Yassitepe E., Buin A., Hoogland S., Sargent E. (2015). Quantum-dot-in-perovskite solids. Nature.

[49] Yin R., Wu R., Miao W., Wang K., Sun W., Huo X., Sun Y., You T., Hao W., Yin P. (2024). Enhanced anchoring enables highly efficient and stable inverted perovskite solar cells. Nano Energy.

[50] Liu N., Liu Z., Wang J., Ye Q., Wang Y., Han W., Xu W., Zhang J., Huang L., Hu Z. (2024). Synchronous surface reconstruction and grain boundary healing toward efficient and stable inverted CsPbI_3_ perovskite solar cells. Chem. Eng. J..

[51] Gong H., Song Q., Zhu T., Zhang C., Huang X., Jing X., You F., Liang C., He Z. (2024). Forming enlarged grain and fixed boundary via a two-step surface modification to achieve stable inverted perovskite solar cells. Chem. Eng. J..

[52] Li Y., Li S., Zhu Z., Li X., Li J., Zhang Q. (2022). Constructing a hybrid high-performance photocatalyst by selective laser precisely heating in nanoscale. Appl. Surf. Sci..

[53] Li Y., Li S., He C., Zhu C., Li Q., Li X., Liu K., Zeng X. (2021). Selective laser-induced preparation of metal-semiconductor nanocomposites and application for enhanced photocatalytic performance in the degradation of organic pollutants. J. Alloys Compd..

[54] Zhao Z., Wang W., Zhou X., Ni L., Kang K., Lee T., Han H., Yuan H., Guo C., Wang M. (2020). Crystal size effect on carrier transport of microscale perovskite junctions via soft contact. Nano Lett..

[55] Yu H., Zhao W., Ren L., Wang H., Guo P., Yang X., Ye Q., Shchukin D., Du Y., Dou S. (2020). Laser-generated supranano liquid metal as efficient electron mediator in hybrid perovskite solar cells. Adv. Mater..

[56] Li C., Yao D., Dong P., Tang Z., Li Y., Chen B., Tian N., Zheng G., Peng Y., Long F. (2024). Synergetic modification on buried and upper surfaces of perovskites with nitrogen-doped carbon quantum dots for efficient and stable solar cells. Appl. Surf. Sci..

[57] Pyatenko A., Wang H., Koshizaki N., TsujI T. (2013). Mechanism of pulsed laser interaction with colloidal nanoparticles. Laser Photonics Rev..

[58] Li X., Pyatenko A., Shimizu Y., Wang H., Koga K., Koshizaki N. (2011). Fabrication of crystalline silicon spheres by selective laser heating in liquid medium. Langmuir.

[59] Li S., Li Y., Liu K., Chen M., Peng W., Yang Y., Li X. (2021). Laser induced core-shell liquid metal quantum dots for high-efficiency carbon-based perovskite solar cells. Appl. Surf. Sci..

[60] Li S., He Z., Li Y., Liu K., Chen M., Yangb Y., Li X. (2021). Laser induced anti-solvent carbon quantum dots in defect passivation for effective perovskite solar cells. J. Alloys Compd..

[61] Li S., Li Y., Liu K., Chen M., Peng W., Yang Y., Li X. (2021). Laser fabricated carbon quantum dots in anti-solvent for highly efficient carbon-based perovskite solar cells. J. Colloid Interf. Sci..

[62] Li S., Li Y., Liu K., Chen M., Peng W., Zhang C., Yang Y., Li X. (2022). Laser generated WS_2_ quantum dots for effective charge transport in high-performance carbon-based perovskite solar cells. J. Power Sources.

[63] Hongsith K., Subtim N., Aryaruknukul A., Panyathip R., Bumrungsan W., Sucharitakul S., Phadungdhitidhada S., Choopun S. (2022). Nickel compound quantum dots as inorganic hole transporting layer in perovskite solar cells. J. Alloys Compd..

[64] Nie J., Niu B., Wang Y., He Z., Zhang X., Zheng H., Lei Y., Zhong P., Ma X. (2023). Multi-functional MXene quantum dots enhance the quality of perovskite polycrystalline films and charge transport for solar cells. J. Colloid Interf. Sci..

[65] Zhao W., Guo P., Liu C., Jia N., Fang Z., Ye L., Ye Q., Xu Y., Glotov A., Novikov A. (2023). Laser Derived Electron Transport Layers with Embedded p-n Heterointerfaces Enabling Planar Perovskite Solar Cells with Efficiency over 25%. Adv. Mater..

[66] Chen J., Ye L., Wu T., Hua Y., Zhang X. (2024). Band engineering of perovskite quantum dot solids for high-performance solar cells. Adv. Mater..

[67] Zhan C., Luo C., Gao F., Wang X., Li Y., Zhao Q. (2024). Indium tin oxide induced internal positive feedback and indium ion transport in perovskite solar cells. Angew. Chem. Int. Edit..

[68] Yu L., Guo T., Yuan H., Zhang Z., Deng Z., Zhao R., Zheng M., Zhang J., Xu W., Liu X. (2021). Effective lewis base additive with S-donor for efficient and stable CsPbI_2_Br based perovskite solar cells. Chem. Eng. J..

[69] Teale S., Degani M., Chen B., Sargent E., Grancini G. (2024). Molecular cation and low-dimensional perovskite surface passivation in perovskite solar cells. Nat. Energy.

[70] Gong C., Li H., Wang H., Zhang C., Zhuang Q., Wang A., Xu Z., Cai W., Li R., Li X. (2024). Silver coordination-induced n-doping of PCBM for stable and efficient inverted perovskite solar cells. Nat. Commun..

[71] Zhao W., Guo P., Wu J., Lin D., Jia N., Fang Z., Liu C., Ye Q., Zou J., Zhou Y. (2024). TiO_2_ electron transport layer with p-n homojunctions for efficient and stable perovskite solar cells. Nano Micro Lett..

[72] Wang W., Holzhey P., Zhou N., Zhang Q., Zhou S., Duijnstee E., Rietwyk K., Lin J., Mu Y., Zhang Y. (2024). Water-and heat-activated dynamic passivation for perovskite photovoltaics. Nature.

[73] Lin Y., Yang V., Cao X., Dasgupta A., Oliver R., Ulatowski A., Mccarthy M., Shen X., Yuan Q., Christoforo M. (2024). Bandgap-universal passivation enables stable perovskite solar cells with low photovoltage loss. Science.

[74] Zhao K., Liu Q., Yao L., Değer C., Shen J., Zhang X., Shi P., Tian Y., Luo Y., Xu J. (2024). Peri-Fused polyaromatic molecular contacts for perovskite solar cells. Nature.

[75] Jiang Q., Zhao Y., Zhang X., Yang X., Chen Y., Chu Z., Ye Q., Li X., Yin Z., You J. (2019). Surface passivation of perovskite film for efficient solar cells. Nat. Photonics.

[76] Wang Z., Ma T., Wang J., Zhu S., Zhang M., Guo M. (2024). Surface passivation for efficient and stable perovskite solar cells in ambient air: The structural effect of ambient molecular. Ceram. Int..

[77] Zhang Z., Chen W., Jiang X., Cao J., Yang H., Chen H., Yang F., Shen Y., Yang H., Cheng Q. (2024). Suppression of phase segregation in wide-bandgap perovskites with thiocyanate ions for perovskite/organic tandems with 25.05% efficiency. Nat. Energy.

[78] Xiao Y., Yang X., Zhu R., Snaith H. (2024). Unlocking interfaces in photovoltaics. Science.

[79] Song Y., Lan S., Yang B., Zheng Y., Zhou Z., Nan C., Lin Y. (2024). High-entropy design for 2D halide perovskite. J. Am. Chem. Soc..

[80] Xiao T., Hao M., Duan T., Li Y., Zhang Y., Guo P., Zhou Y. (2024). Elimination of grain surface concavities for improved perovskite thin-film interfaces. Nat. Energy.

[81] Rong Y., Hu Y., Mei A., Tan H., Saidaminov M.I., Seok S.I., McGehee M.D., Sargent E.H., Han H. (2018). Challenges for commercializing perovskite solar cells. Science.

